# Resting-state frontal, frontlateral, and parietal alpha asymmetry: A pilot study examining relations with depressive disorder type and severity

**DOI:** 10.3389/fpsyg.2023.1087081

**Published:** 2023-03-07

**Authors:** Gabriela M. Marcu, Raluca D. Szekely-Copîndean, Ana-Maria Radu, Mihaela D. Bucuță, Radu S. Fleacă, Ciprian Tănăsescu, Mihai D. Roman, Adrian Boicean, Ciprian I. Băcilă

**Affiliations:** ^1^Department of Psychology, Lucian Blaga University of Sibiu, Sibiu, Romania; ^2^Faculty of Medicine, Carol Davila University of Medicine and Pharmacy, Bucharest, Romania; ^3^Scientific Collective for Research in Neuroscience, Clinical Psychiatric Hospital “Dr. Gh. Preda”, Sibiu, Romania; ^4^Department of Social and Human Research, Romanian Academy, Cluj-Napoca, Romania; ^5^Center for Psychological Research, Lucian Blaga University of Sibiu, Sibiu, Romania; ^6^Faculty of Medicine, Lucian Blaga University of Sibiu, Sibiu, Romania

**Keywords:** alpha asymmetry, depression, biomarker, treatment-resistant depression, resting state EEG, depression severity, regional asymmetries

## Abstract

**Introduction:**

The search for biomarkers has been central to efforts of improving clinical diagnosis and prognosis in psychopathology in the last decades. The main approach has been to validate biomarkers that could accurately discriminate between clinical diagnoses of very prevalent forms of psychopathology. One of the most popular electrophysiological markers proposed for discrimination in depressive disorders is the electroencephalography (EEG)-derived frontal alpha asymmetry. However, the validity, reliability and predictive value of this biomarker have been questioned in recent years, mainly due to conceptual and methodological heterogeneity.

**Methods:**

In the current non-experimental, correlational study we investigated relationship of resting-state EEG alpha asymmetry from multiple sites (frontal, frontolateral, and parietal) with different forms of depressive disorders (varying in type or severity), in a clinical sample.

**Results:**

Results showed that alpha asymmetry in the parietal (P3-P4) was significantly higher than in the frontal (F3-F4) and frontolateral sites (F7-F8). However, we did not find significant relations between alpha asymmetry indices and our depressive disorder measures, except for a moderate positive association between frontolateral alpha asymmetry (eyes-closed only) and depressive disorder severity (determined through clinical structured interview). We also found no significant differences in alpha asymmetry between participants, depending on their depression type.

**Discussion:**

Based on results, we propose the parietal and frontolateral asymmetry indices to form hypotheses that should not be abandoned in the depression markers research, but worth for further experimental research. Methodological and clinical implications of the current findings are discussed.

## Introduction

The relation between electroencephalography (EEG), frontal alpha asymmetry (fAA), and psychopathology has been intensely investigated in the last decade (Allen et al., 2018). The primary goal of such research has been to establish whether fAA could be a reliable predictor and/or biomarker of very prevalent forms of psychopathology such as depression (e.g., NIMH Strategic Plan on Research Domain Criteria and termed Precision Medicine). While early research indicated fAA as a promising diagnostic tool for depression (Henriques and Davidson, 1990, 1991; Bruder et al., 1997), more recent approaches have pointed out multiple obstacles that need to be surpassed before establishing any reliable link between fAA and depression (Smith et al., 2017). Some of the most important obstacles refer to methodological issues in extracting and computing fAA (Smith et al., 2017) on the one hand, and the fact that psychopathology such as major depressive disorder (MDD) is very heterogeneous (Coan et al., 2006), on the other hand. Recent large-scale investigations (Metzen et al., 2022) found considerable inconsistencies between frontal alpha asymmetry studies in depression while new often-neglected asymmetry markers were proposed for study, such as frontolateral alpha asymmetry (flAA) (Lopez-Duran et al., 2012; Monni et al., 2022) or parietal alpha asymmetry (pAA) (Stewart et al., 2011; Metzen et al., 2022).

The current pilot study investigated the relation between alpha asymmetry indices at different sites (frontal, frontolateral, and parietal) and MDD by tackling some of the methodological issues that have been pointed out in the literature. In a non-experimental correlational design, the relationship between depression scores and the magnitude of asymmetry in different homologous locations was measured and tested for predictive value on a clinical sample.

The reliability of the fAA might depend on the signal acquisition protocol, with eye position and recording duration being among the most relevant aspects. In the case of eye position, resting-state signal acquisition in an eyes-closed condition was found to lead to more reliable fAA indices, relative to an eyes-open condition, across multiple measurements and equipment (Metzen et al., 2022). However, there are no recommendations to favor the former condition, and many studies that have integrated both conditions have failed to find significant differences between the two conditions (Reid et al., 1998; Gordon et al., 2010; Stewart et al., 2010). Moreover, such studies have pointed out that in the eyes-open condition, relative to the eyes-closed condition, any existing alpha asymmetry is still noticeable, even though alpha may be suppressed. In the case of duration of signal acquisition, in order to obtain reliable fAA indicators, a minimum of 8 min is recommended (Hagemann, 2004).

Another issue impacting the reliability of fAA refers to signal processing – the chosen referencing method. Among the most used online references are Cz electrode location (standard 10–20 system), average reference (AV) or linked mastoids (LE); because of distortions induced by volume conduction and by reference electrodes the signal can further undergo a spatial transformation to enhance the spatio-temporal resolution. Current-source density is one such transformation that uses mathematical algorithms to spatially represent the local effects of the brain sources responsible for the scalp-recorded potentials (Burle et al., 2015; Kamarajan et al., 2015). While there is no consensus on which referencing method is best, using a current-source density transformation can effectively reduce spatial noise, especially in the low-frequency part of the potential (Wójcik, 2013). Its use for measuring validity of fAA was confirmed in both resting-state and task-eliciting conditions (Stewart et al., 2014), and when paired with a latent factorial approach (Monni et al., 2022). Stewart et al. (2014) and Smith et al. (2017) recommend using current-source density transformation in alpha oscillations research because compared to other montages (Cz, LE or AV), it better attenuates the contribution of distal references to surface potential. Given its capacity to eliminate non-frontal alpha power from further analysis (Smith et al., 2017), the current-source density derivation may be a better choice for computing alpha asymmetry (Hagemann and Naumann, 2001).

Since alpha asymmetry is derived from EEG recordings, decisions about signal cleaning may impact alpha asymmetry estimates as well. For instance, decisions about removal or correction of very common ocular and muscle movement artifacts from frontal electrodes may affect alpha asymmetry computations, which are often based on frontal area recordings. To eliminate such artifacts, researchers may opt to either visually inspect the recording and simply eliminate segments contaminated by eye activity, or to use an automatic algorithm. Even though it has been argued to be robust against resting-state alpha asymmetry miscalculations (Hagemann and Naumann, 2001), the main limitations of the artifact removal approach are potentially missing certain artifacts during visual inspection and losing data through segment removal (epoch rejection). The alternative to epoch rejection is the independent component analysis (ICA) correction, or blind-source separation techniques, followed by automatic artifact correction algorithms to classify the components to mark them for removal (Delorme et al., 2007). Additionally, deciding to use an ICA approach involves further choices, such as the selection of unmixing algorithms that least influence the distribution of alpha activity (Smith et al., 2017).

Calculation of alpha asymmetry could follow two approaches, either the use of conventional alpha band, or the use of an individualized interval derived from the individual EEG data. The last approach is thought to have more measurement sensitivity, which is increased by individualizing the alpha band (calculating its bandwidth relative to individual alpha peak frequency). Individual variations are reflected both by the individual Alpha Peak frequency (iAPF) (Klimesch et al., 1993) and by the alpha bandwidth, which in turn can be divided into several subdivisions on which asymmetry indices can subsequently be calculated. However, comparing these subdivisions in homologous frontal locations does not seem to come with a greater sensitivity than that of the asymmetry calculated on the conventional alpha band (Segrave et al., 2011). In addition, the measurements by individualized alpha frequency peak windows appear to be closely correlated with those of fixed frequency band, without bringing any additional reliability or validity to the calculation of fAA (Zhang and Allen, 2023), these results methodologically support any of the two approaches to alpha frequency, in fAA measurement.

Stability of a proposed (physiological) index is a necessary condition for using it as a reliable biomarker. Alpha asymmetry has been found to be a relatively stable coefficient in healthy individuals, leading to its assimilation with a predisposition or trait (Allen et al., 2004). The stability was also confirmed in clinical populations, in people undergoing depression treatment (van der Vinne et al., 2019) and in people who had been exposed to maltreatment (female sample; Miskovic et al., 2009). Alpha asymmetry also seems to be preserved both in the frontal location (F3-F4) and at the parietal level (P3-P4) (Schmidt et al., 2022).

Once the measurements and stability requirements are met, the alpha asymmetry as biomarker should function as a reliable index of relative alpha band activity between the brain hemispheres and discriminatively reflect different psychological states. Different models were proposed for interpreting the index; one is the dispositional model of asymmetry that linked the right/left lateralization with negative/positive emotion (Davidson, 2004). More recently, an approach-withdrawal model (Coan and Allen, 2004) proposed further interpretations of the index as the anticipation of the brain’s response to affectively charged stimuli (Sutton and Davidson, 2000), or of suicidal behavior, when low alpha was considered (Jang et al., 2020).

The direction of the asymmetry is not currently established for depression. Existing literature has suggested a combined effect of age, sex, and intensity of the pathology both on the positive and negative value of the index. The relationship between fAA and affective psychopathology is usually interpreted in the key of an inverse relationship between alpha power and cortical activity (Coan and Allen, 2004), a higher value of alpha amplitude/power reflecting less cortical activity and vice versa. In some studies, relatively higher alpha band power in the left vs. right frontal channels (left-sided fAA) was reported in subjects with MDD compared to healthy individuals (Henriques and Davidson, 1990, 1991; Allen et al., 2004), while in other studies that examined age and gender, opposite results were obtained, such as right-sided fAA, for certain age categories and gender (van der Vinne et al., 2017). Some researchers even considered just the absolute value of the asymmetry as a relevant marker (Gollan et al., 2014).

Indices of frontal and parietal alpha asymmetry appear to be more commonly used in association with affective disorders and psychopathology in general. Fewer results were reported on frontolateral asymmetry which, associated with prefrontal control, was most investigated in disorders involving self-control dysfunctions, such as panic disorder (Thoma et al., 2021), suicidal behavior (Park et al., 2019), or to differentiate stress reactions (Quaedflieg et al., 2015). The discriminative capacity of the flAA (F7-F8) in depression was found to be less reliable than that of the fAA (F3-F4) (Jaworska et al., 2012), which partly explains whythis index was less researched in relation to depression and with the approach/avoidance motivation model. Parietal alpha asymmetry was found to display a distinct involvement in emotional processing or arousal (Stewart et al., 2011; Koller-Schlaud et al., 2020), both of which related with depressive mood (Scibelli et al., 2016; Zhang et al., 2019; Gray et al., 2020). The parietal cortex was proposed as a valuable candidate for study (Stewart et al., 2011), due to its reported implication in depression-related attention and executive function deficits. Given these numerous findings, other asymmetry sites, like the frontolateral (Lopez-Duran et al., 2012) and parietal (Metzen et al., 2022), have been proposed for investigation in relation to depression, in addition to frontal asymmetry. In the last 50 years, the frontal alpha asymmetry was the most popular electrophysiological measure of depressive disorders. Still, the heterogeneity of the MDD (difference severity levels and comorbidity with anxiety) raised questions about how different diagnosis subcategories compare, in terms of being predicted by the asymmetry index. Having a marker that is sensitive to conditions like severity or comorbidity is important for clinical use; therefore, several studies investigated the impact of comorbidities on index value (Nusslock et al., 2018; Horato et al., 2022). Findings showed either no differences in alpha lateralization in co-morbid and depressed individuals (Thibodeau et al., 2006), or different lateralization directions: greater activation over right than left anterior and posterior sites in anxious depressed patients (Bruder et al., 1997) or slightly greater left than right (Horato et al., 2022). In one study, comorbid anxiety appeared correlated with the biggest relative change in frontal alpha asymmetry compared with depression without comorbid anxiety (Mathersul et al., 2008), while in another study, frontal asymmetry appeared to be predicted by anxiety only (Adolph and Margraf, 2017). A recent meta-analysis concluded that EEG alpha asymmetry continues to have an unclear validity as a biomarker for depression, while being more robust in anxiety (Kaiser et al., 2018).

The lack of a procedural consensus regarding the conceptualization and measurement of alpha asymmetry, as well as the heterogeneity of the diagnostic subcategories of depression, cause inconsistencies that persist in the attempts to validate fAA as a biomarker for depressive pathology. The present study uses the methodological decisions for asymmetry scores calculations, electrode sites, referencing choices, and artifact removal that are most validated in literature, to identify conditions in which the alpha asymmetry index has the greatest potential for discrimination.

## Methods

### Participants

Twenty-four participants (13 females, 11 males), with ages ranging from 21 years to 69 years (*M*_age_ = 45.33, *SD* = 14.63), were recruited for this study. Participants were former or current patients at a local psychiatric hospital in Romania and had been previously diagnosed with depressive disorder by a hospital psychiatrist. Participants met the criteria for different forms of depressive disorder as follows: 13 were diagnosed with recurrent depressive disorder (RDD), 6 with major depressive disorder (MDD), 4 with mixed anxiety and depressive disorder (MADD) and 1 with severe depressive disorder (SDD). Also, the sample contained a sub-group of 16 participants (8 females, 8 males) that met the criteria for treatment-resistant depression. The criteria refer to not obtaining an adequate therapeutic response after the administration of two antidepressants, from different pharmacological classes, in adequate therapeutic doses, for a minimum of 6 weeks. All participants were right-handed and had no known cognitive disorders, as screened by the hospital’s clinical psychologist. Written informed consent was collected from all the participants and all the procedures complied with the AMA’s Declaration of Helsinki recommendations for medical research involving human subjects.

### Procedure

#### EEG data acquisition

The resting state EEG signal was recorded using a 34-sintered Ag/AgCl electrode (including A1, A2, Oz, FPz and Gnd) BeeMedic System (Comby Cap and NeuroampII5S + x39 amplifier). Sampling rate was set to 500 Hz and impedance was kept below 10 kΩ (Allen et al., 2004). The EEG recording lasted 20 min, beginning with a 10-min eyes-open (EO) condition, followed by a 10-minute eyes-closed (EC) condition. Recordings took place in a dimly lit room. Participants were invited to adopt a comfortable position in the provided armchair and try to abstain from any movements as much as possible. To minimize potential muscle tension artifacts, a chest belt was also used.

### Data preprocessing

The WinEEG software was used for preprocessing. The preprocessing pipeline was the same for all participants. First, 19 channels were selected from 32, according to the 10–20 system, and the recording was downsampled to 250 Hz. Continuous EEG signal was 0.1 Hz high pass filtered and then 30 Hz low pass filtered (Luck, 2014), to effectively reduce superimposed artifacts from sources like line noise (50 Hz) or other non-physiological interferences. Filtered signal was segmented into 4-s epochs (50% overlap, Hann window). As no standard algorithm is mentioned in the literature for channel rejection, we chose to keep only recordings with a maximum of one channel rejected. Rejection was based on identification of bad channels (channels that do not fluctuate with the others) during acquisition.

To remove ocular (EOG), myographic (EMG) and cardiac artifacts, a second-order blind identification (SOBI) algorithm of ICA was used. Research on denoising electroencephalography has emphasized that it is a method that is based on second-order statistics, and unlike other ICA methods that employ higher-order statistics, is faster, more reliable and reproducible (Urigüen and Garcia-Zapirain, 2015). Moreover, SOBI does not require using a reference channel, being especially useful when one such channel is not available (e.g., EOG) and outperforms other artifact-removal methods by having a reduced distortion of all frequency bands (Joyce et al., 2004; Liu and Zhang, 2022). In the current sample, a maximum of two components per participant (corresponding to either eye-movement or EMG artifacts) were rejected. EEG recordings were visually inspected and segments with strong artifacts were marked for rejection. Channel interpolation was applied (if any “bad” channel was marked) and the EEG signal was re-referenced to current source density (CSD) to improve its spatial resolution (Burle et al., 2015) by facilitating spatial separation of temporally overlapping components (Kamarajan et al., 2015).

### EEG data analysis

The processed EEG recordings were imported in NeuroGuide (Applied Neuroscience ltd.) and an automatic artifact rejection was run (Z Score Artifact Rejection for “high” eye movement and drowsiness selection with setting of 1.5 standard deviation threshold for the Amplitude Multiplier) followed by a visual inspection of the whole recording to determine whether the parameters were effective and removal of segments of data that were not identified with the automatic parameters. The “clean” EEG recording was checked to be at least 8 min (Allen et al., 2004) and the Split Half and Test–retest values to be over 0.9. For further analysis, we chose to use surface Laplacian montage. This allowed us to get a valid and reliable separation of EEG spectra frequency bands like theta (4–8 Hz) and alpha (8–13 Hz) (Kayser and Tenke, 2015), while having a less sharp but more distributed topography. It also allowed us to diminish effects attributed to volume conduction, reference location or computation of an average reference, and to easily identify focal abnormal brain activity.

### Regional alpha asymmetry calculation

Power spectrum was calculated using the Fast Fourier transformation (FFT) and averaged for each condition. Conventional FFT-derived alpha power (8–12 Hz) was selected from the generated QEEG report (Neuroguide software) at the three target-paired sites: F3-F4, F7-F8, and P3-P4, and regional alpha asymmetry (mid-frontal, lateral frontal, parietal) was calculated from natural-log transformed EEG alpha power in the left hemisphere from natural-log transformed power at homologous sites in the right hemisphere, for both eyes-open and eyes-closed conditions.

### Quantitative EEG analysis of resting alpha asymmetry

Alpha asymmetry was analyzed at anterior sites (mid-frontal, lateral frontal), and posterior (parietal) sites. For the three regions (mid-frontal, lateral-frontal, parietal) average values were calculated for groups of different conditions and topographic distributions were generated, prior to further statistical analyses. Quantitative EEG analyses were performed with the NeuroStat program (Applied Neuroscience Inc.)

### Depression evaluation

#### Montgomery–Åsberg depression rating scale

The Montgomery–Åsberg Depression Rating Scale (MADRS) a 10-item diagnostic questionnaire (range 0–60) was used to measure the severity of depressive episodes (Montgomery and Asberg, 1979).

#### Beck depression inventory-II

The Romanian version of the Beck Depression Inventory (i.e., BDI-II) was used to assess depression. The BDI-II is a 21 items self-rating scale, with items being rated on a 4-point scale (David et al., 2012).

#### MDD total

Patients were diagnosed by psychiatrists using the Structured Clinical Interview for DSM 5 for major depressive episodes and for secondary diagnosis of anxiety. A total MDD score was calculated by adding the BDI II and MADRS scores, for further analyses.

### Statistical analyses

Pearson zero-order correlations were computed to examine relations between depressive disorder scores and alpha asymmetry values. A two-way repeated measures analysis of variance was used to test differences in alpha asymmetry between condition (eyes-open, eyes-closed) and location (frontal, frontolateral, parietal). A mixed analysis of variance was used to examine differences in alpha asymmetry values between participants with and without treatment-resistance diagnosis and location (collapsed across conditions). Similarly, a mixed analysis of variance was used to examine alpha asymmetry differences between different forms of depressive disorder and location. For the latter analysis, we excluded one participant, the only one diagnosed with severe depressive disorder, to make groups more comparable. All analyses were run in R programming language version 4.2.1 (R Core Team, 2022). Data and analysis code are publicly available on the Open Science Framework.^1^

## Results

Table 1 displays descriptive statistics, as well as correlations between each measure of depressive disorder and each alpha asymmetry value for the three locations (frontal, frontolateral and parietal) and the two conditions (eyes-open, eyes-closed). Results showed a significant positive correlation between MADRS score only with frontolateral alpha asymmetry, and only when participants were in the eyes-closed condition (see Table 1). Moreover, alpha asymmetry scores were significantly correlated between conditions within each location (Table 1). BDI and total MDD scores were not significantly correlated with any of the alpha asymmetry measures.

**Table 1:** Means, Standard Deviations, and Correlations Between Measures.

Variables	1	2	3	4	5	6	7	8	9
1. MADRS	–								
2. BDI	0.47*	–							
3. MDD _total_	0.84**	0.87**	–						
4. fAA EO	0.08	0.10	0.10	–					
5. fAA EC	0.03	0.01	0.02	0.72**	–				
6. flAA EO	0.28	0.09	0.21	0.31	0.34	–			
7. flAA EC	0.48*	−0.02	0.26	0.47*	0.38	0.41*	–		
8. pAA EO	0.07	0.19	0.15	0.54*	0.46*	0.39	0.34	–	
9. pAA EC	0.10	0.09	0.11	0.47*	0.43*	−0.09	0.38	0.50*	–
*M*	21.25	23.04	44.29	13.70	11.33	5.10	−9.35	29.37	39.37
*SD*	7.18	7.92	12.94	55.51	53.42	63.74	45.89	48.63	67.78

fAA = frontal alpha asymmetry; flAA = frontolateral alpha asymmetry; pAA = parietal alpha asymmetry; EO = eyes-open; EC = eyes-closed. Correlations were computed using Pearson method. Non-significant correlations are displayed in gray.

*p < 0.05, **p < 0.01.

### Contrasting eyes-open and eyes-closed conditions

**Figure 1:**
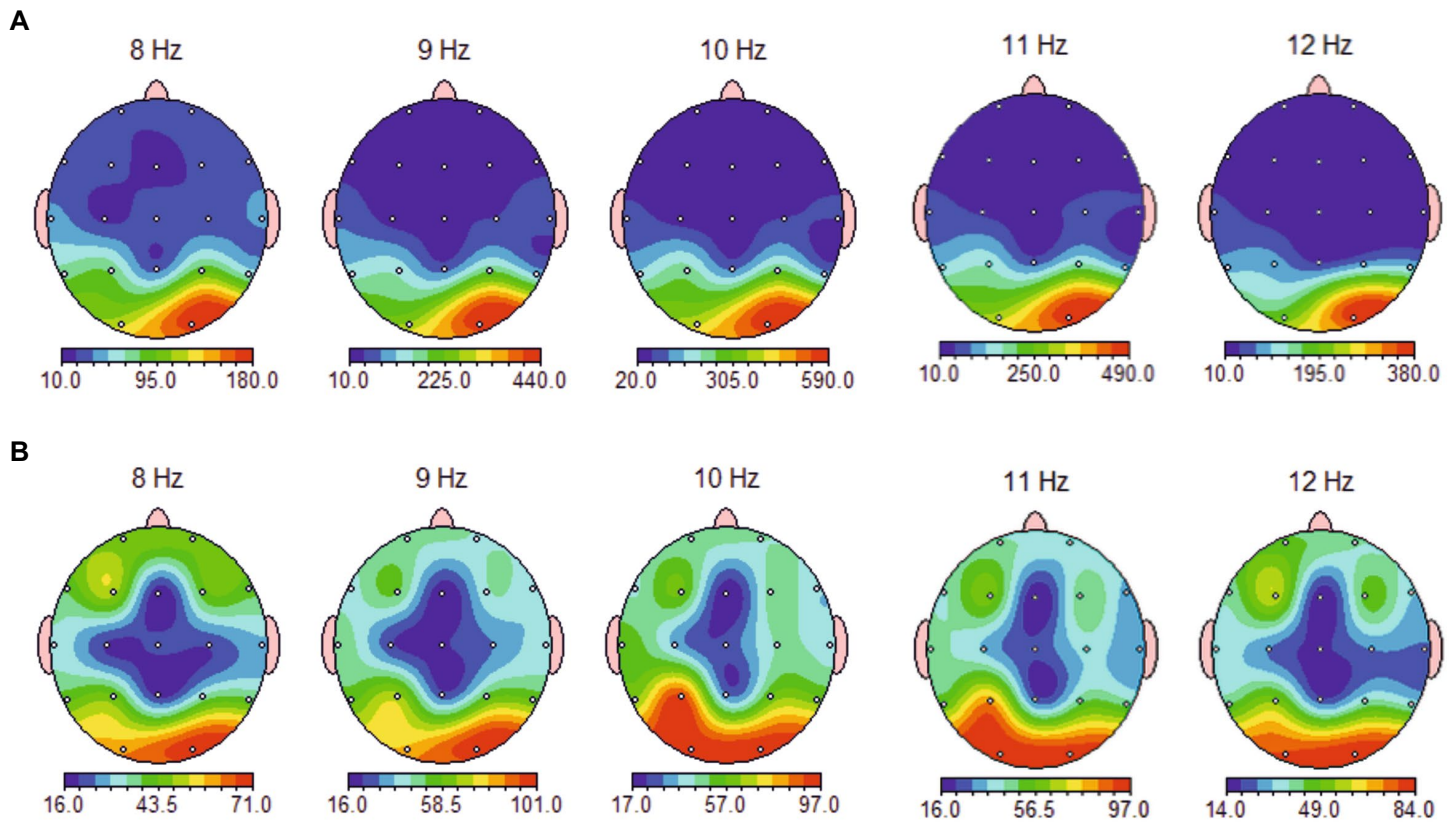
Topographical distribution of 8–12 Hz band average alpha power in eyes-closed **(A)** and eyes-open **(B)** condition, showing no relevant asymmetrical distribution at measured sites (frontal, frontolateral and parietal) and a focal occipital pattern of alpha activation in eyes-closed condition.

**Figure 2:**
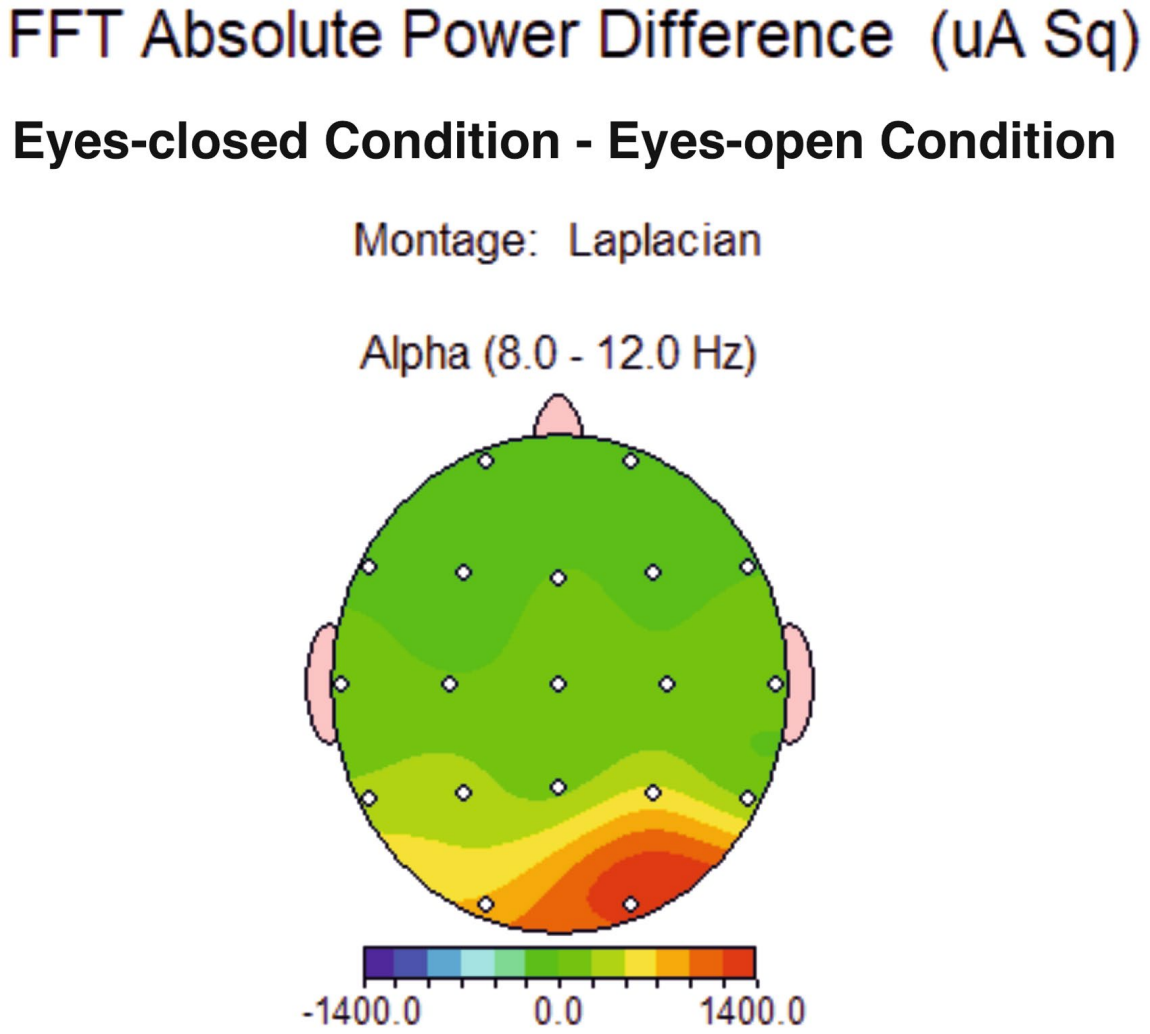
Topographical distribution of conventional alpha band average power difference between eyes-closed and eyes-open condition, showing no difference for the alpha band in the two conditions at measured sites (frontal, frontolateral and parietal).

Spectral maps of eyes-open and eyes-closed conditions for the whole alpha band range (8–12 Hz) revealed no notable asymmetrical distribution across the three locations (frontal, frontolateral, and parietal), neither in the 1 Hz topographical distribution (Figure 1) nor in the aggregated bandwidth (Figure 2).

### Contrasting non-treatment-resistant depression and treatment-resistant depression groups

**Figure 3:**
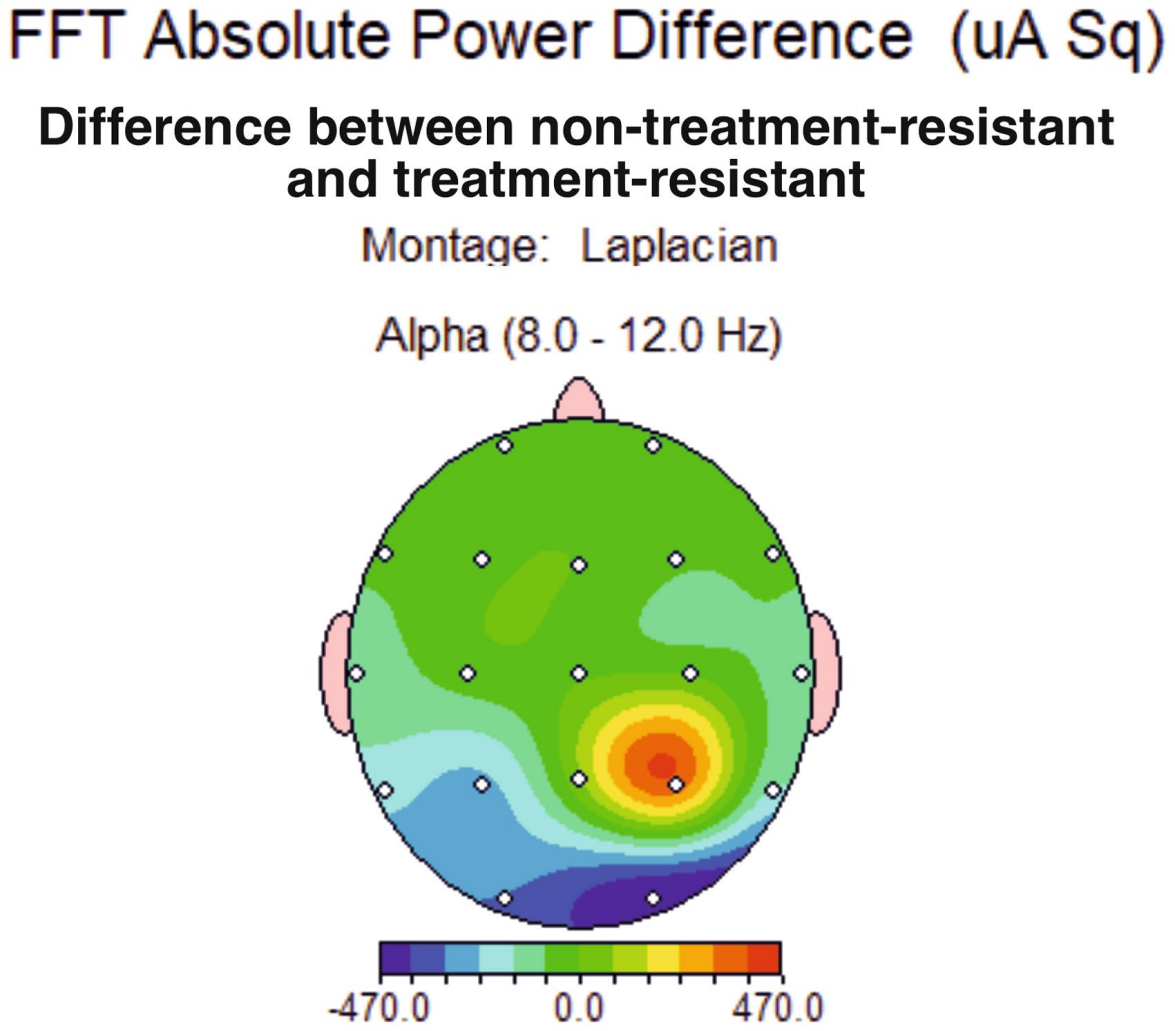
Topographical distribution of conventional alpha band average power difference between major depression group non-treatment-resistant (non-TRD) and treatment-resistant depression group (TRD), showing a slight power difference for the alpha band at P4 site.

The spectral distribution of the alpha band was averaged across groups and then subtracted across all locations (TRD group average values were subtracted from non-TRD group average values). The result showed a parietal alpha asymmetry of 470 uA power (Figure 3), which was further tested for relevance.

**Figure 4:**
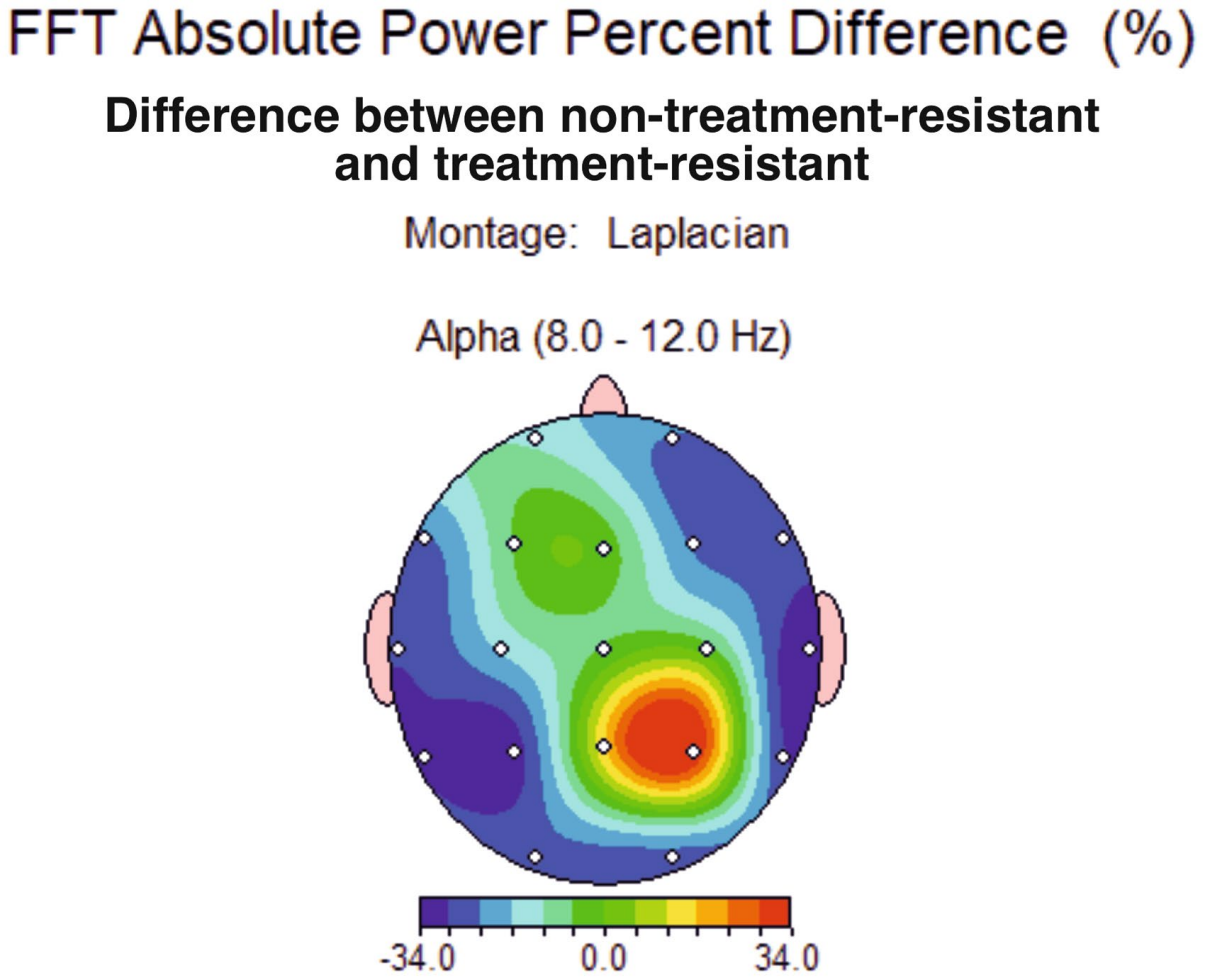
Topographical distribution of conventional alpha band average power percent difference between major depression group non-treatment-resistant (non-TRD) and treatment-resistant depression group (TRD), showing a 34% difference for the alpha band at P4 site.

A percent difference was computed between the groups’ averages and a 34% difference in alpha band power at right parietal (P4) resulted (Figure 4). The statistical analysis found this difference not significant, for the current sample, but it might be considered as starting point in further analyses on larger samples.

**Figure 5:**
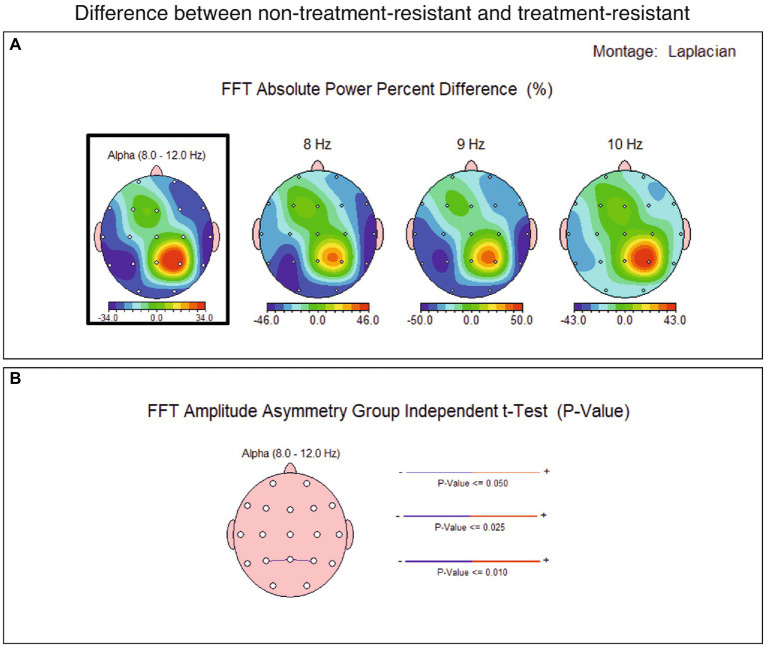
**(A)** Topographical distribution of alpha average power percent difference between major depression group non-treatment-resistant (non-TRD) and treatment-resistant depression group (TRD), showing a 34% difference for the alpha band (8–120 Hz) at P4 site and a maximum difference at 9 Hz: 50% bigger at P4 in the non-TRD group. **(B)** Difference in FFT amplitude asymmetry across all sites between non-TRD and TRD group, showing a slight significant value (*p* < 0,05) at P3-P4 sites in conventional alpha band (bottom). The thin blue line linking P3 and P4 sites is interpreted as smaller alpha asymmetry at the parietal site, for the non-TRD group.

Moreover, the spectral analysis of 1 Hz alpha band sub-divisions indicates a maximum of difference at the frequency of 9 Hz (Figure 5), suggesting that in treatment-compliant, more than in treatment resistant depression, a parietal lower alpha asymmetry might be of interest to further investigate - which is partially consistent with results associating lower alpha at parietal site with depression (Kan and Lee, 2015).

### Contrasting frontal, frontolateral, and parietal alpha asymmetry

**Figure 6:**
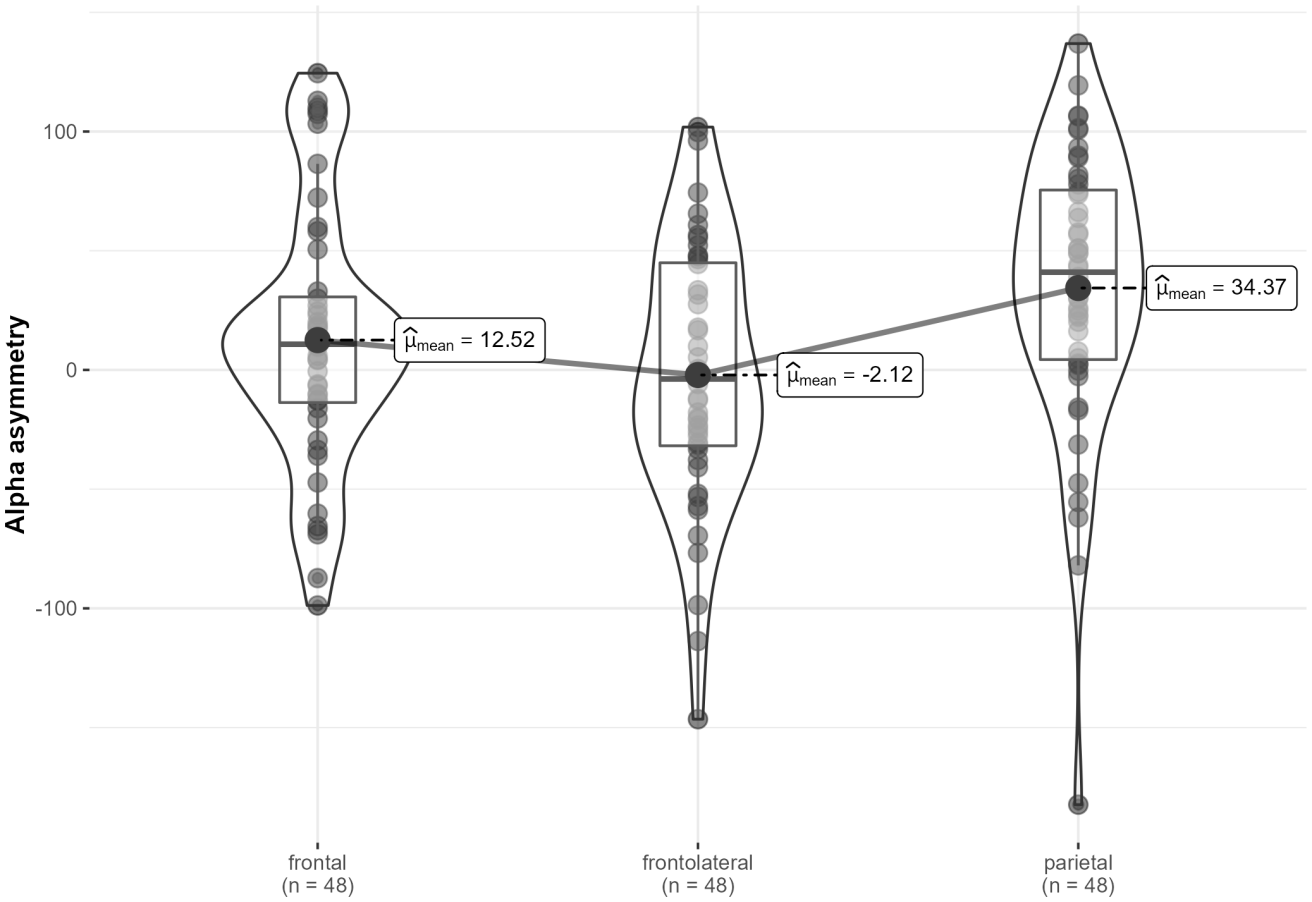
Alpha asymmetry in three locations (frontal, frontolateral and parietal), across conditions.

Across conditions, alpha asymmetry significantly varied between locations, *F* (1.87, 43.01) = 6.17, *p* = 0.005, η^2^_G_ = 0.07 (Figure 6). *Post-hoc* tests showed that frontal asymmetry, *M* = 12.52, *SD* = 53.91, was significantly lower than parietal asymmetry, *M* = 34.37, *SD* = 58.58, *t*_Student_ (47) = −2.6, *p*_Bonferroni_ = 0.038. Moreover, frontolateral asymmetry, *M* = −2.12, *SD* = 54.86, was significantly lower than parietal asymmetry, *t*_Student_ (47) = −3.91, *p* < 0.001. Frontal and frontolateral alpha asymmetries were not significantly different, *t*_Student_ (47) = 1.62, *p* = 0.333.

**Figure 7:**
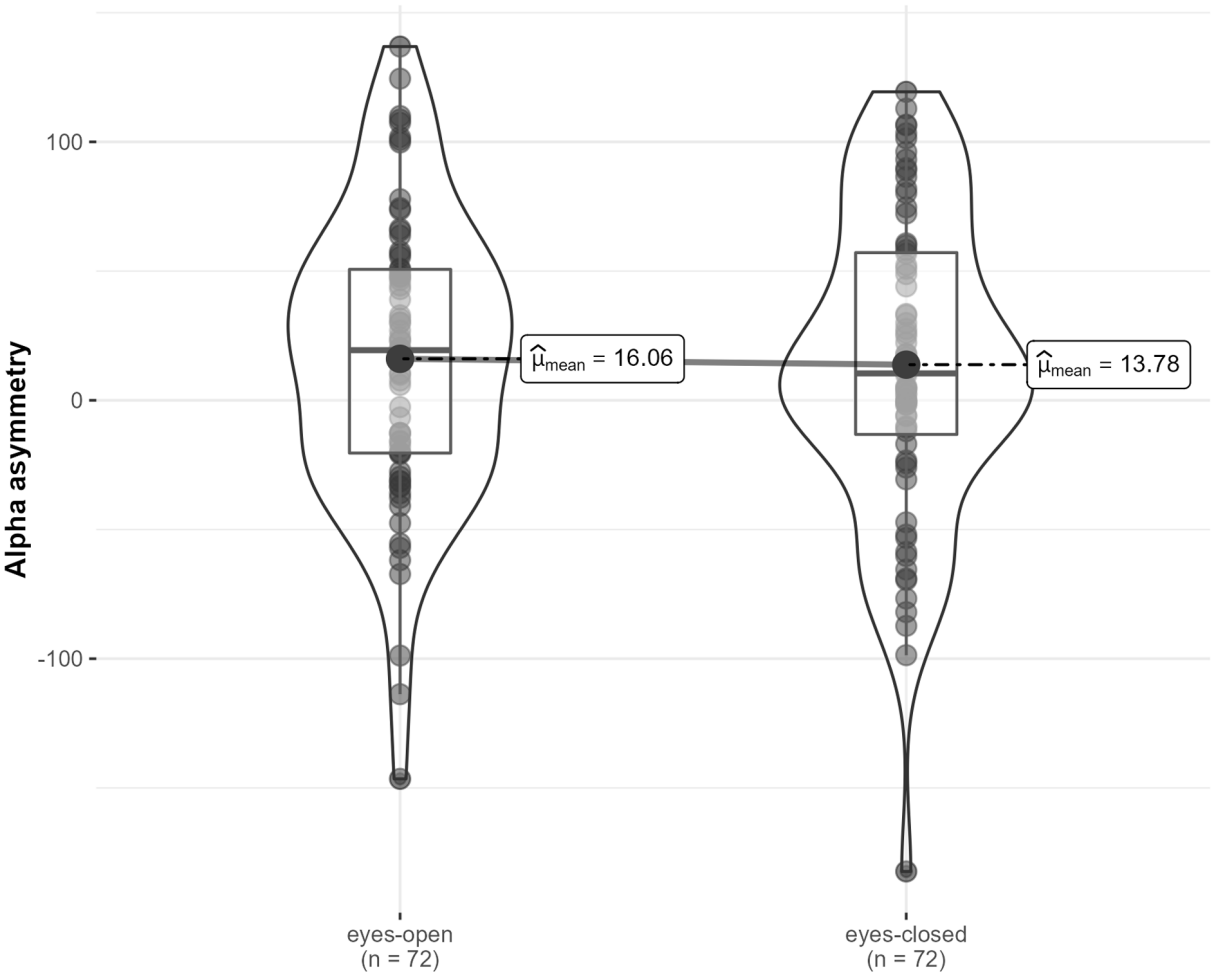
Alpha asymmetry in two conditions (eyes-open, eyes-closed), across locations.

Across locations, there were no significant differences in alpha asymmetry between the eyes-open condition, *M* = 16.06, *SD* = 56.05, and the eyes-closed condition, *M* = 13.78, *SD* = 59.15, *F* (1, 23) = 0.09, *p* = 0.767, η^2^_G_ < 0.001 (Figure 7). There were no significant differences in alpha asymmetry between conditions depending on location, *F* (1.81, 41.55) = 1.48, *p* = 0.240, η^2^_G_ = 0.008.

### Depression diagnosis depending on alpha asymmetry location

**Figure 8:**
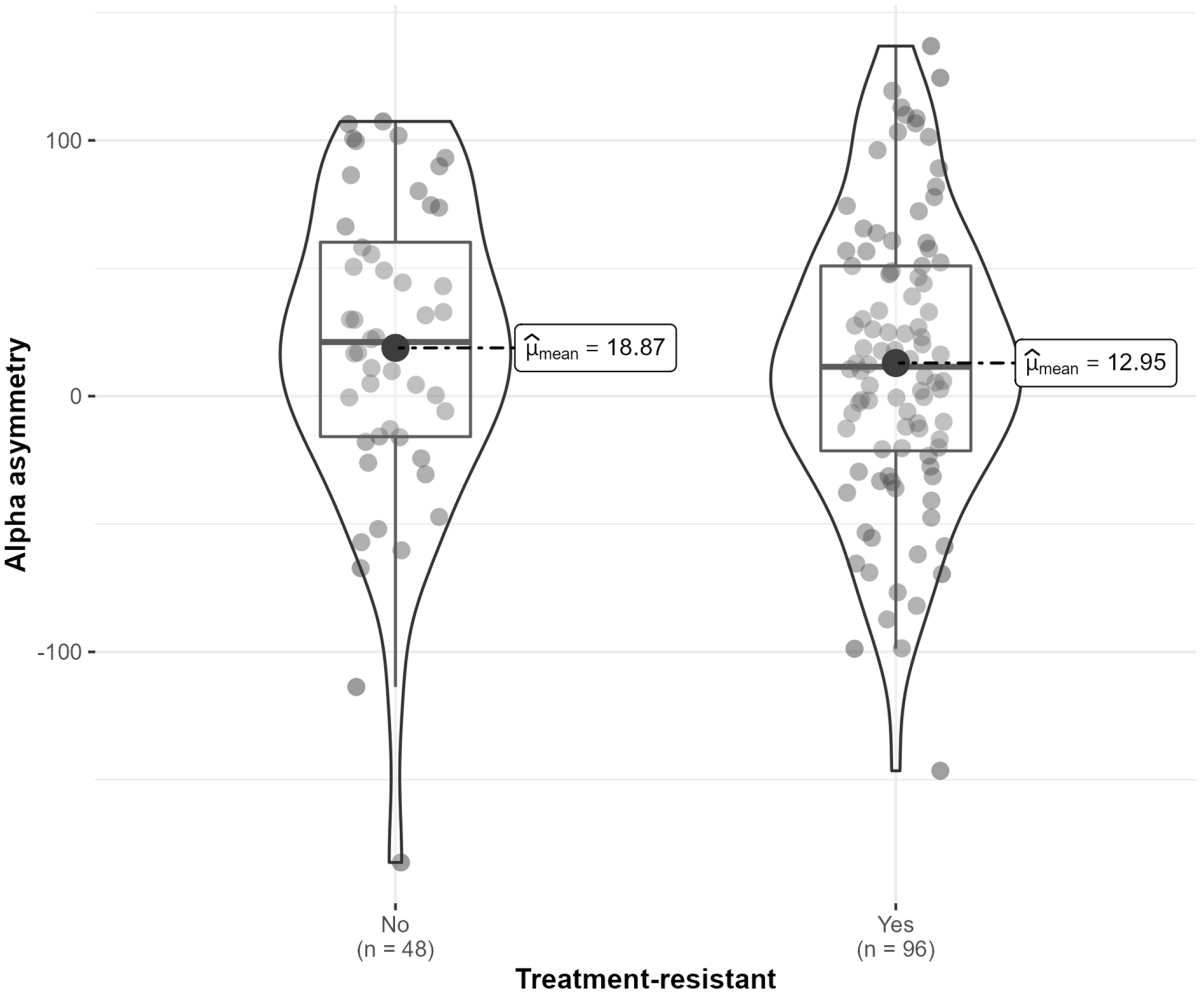
Alpha asymmetry scores distribution for participant with and without treatment-resistant depressive disorder.

Alpha asymmetry was not significantly different for participants diagnosed with treatment-resistant depressive disorder, compared to those that did not have this diagnosis, *F* (1, 22) = 0.12, *p* = 0.735, η^2^_G_ = 0.003 (Figure 8). The interaction with location was also not statistically significant (*p* = 0.896).

**Figure 9:**
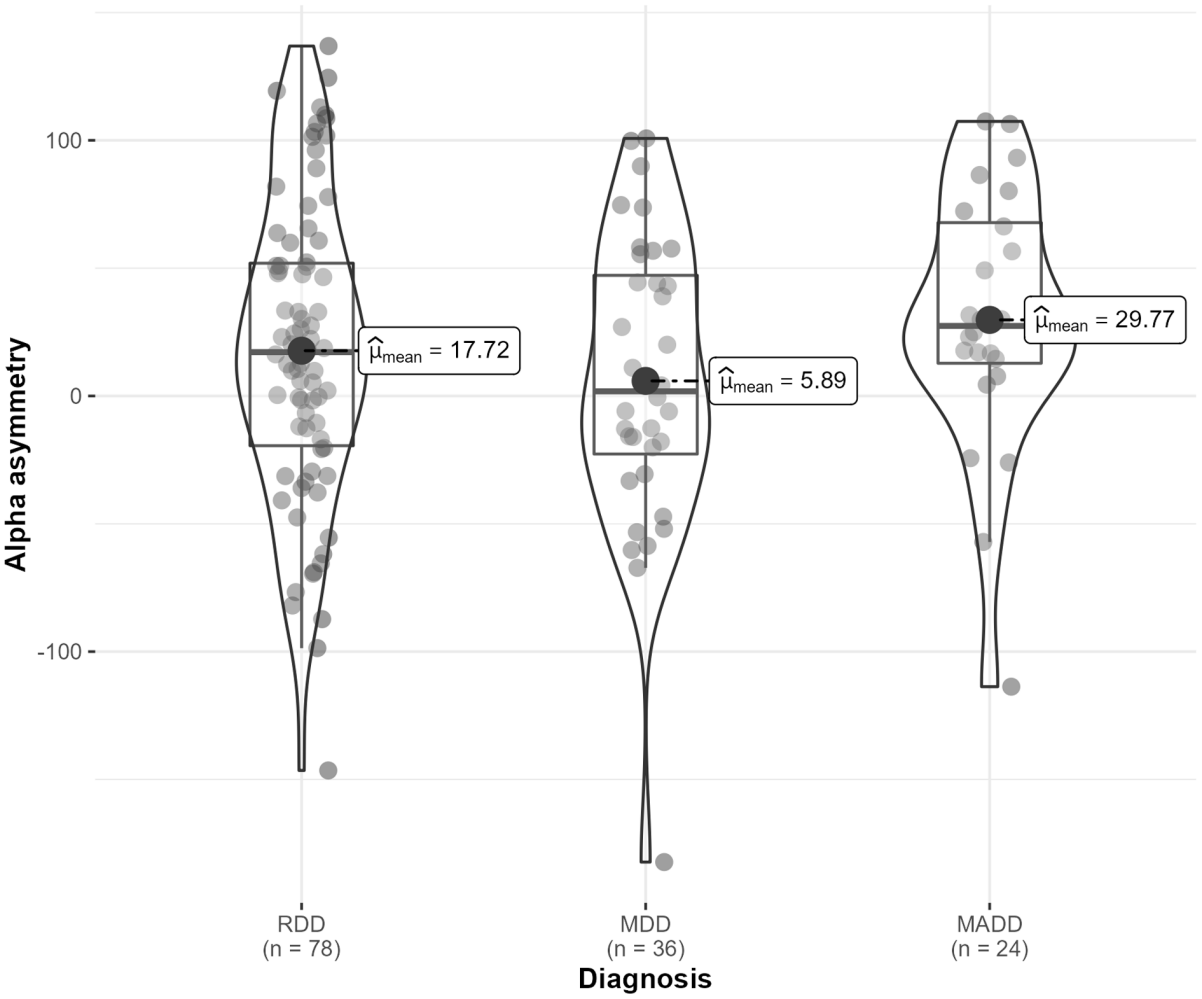
Frontal alpha asymmetry in three diagnostic categories (RDD, MDD, MADD).

Similarly, there were no significant differences in alpha asymmetry depending on depressive disorder diagnosis (i.e., recurrent, major, mixed), *F* (2, 20) = 0.44, *p* = 0.653, η^2^_G_ = 0.027 (Figure 9). The interaction with location was not statistically significant (*p* = 0.362).

## Discussions

Given the large interest in finding and confirming reliable biomarkers for high-prevalence psychopathologies like depression, our study’s primary objective was to explore the current asymmetry predictors for MDD, and to facilitate decision upon hypotheses worth for further investigation in future validation studies. In a non-experimental, correlational study, we explored resting-state associations between three types of EEG alpha asymmetry (frontal, frontolateral and parietal) and a general MDD score, while controlling for conditions (eyes-open and eyes-closed) and for MDD severity and resistance to pharmacological treatment.

The main findings were that the alpha asymmetry is relatively stable in eyes-open and eyes-closed conditions, while it significantly varies between locations across conditions. We could not find reliable links of fAA with depression, and there were no significant differences between types of depression, as far as alpha asymmetry is concerned. The results are to be interpreted considering the study’s limitations — the small sample size (which can lead to false negative results) and the lack of a healthy control group, respectively.

Despite its limitations, the present study contributes to the small body of empirical evidence on alternative asymmetry indexes in MDD. Moreover, the study addresses and implements decisions for EEG data collection and preprocessing that are up-to-date and most agreed upon by the psychophysiological research community Specifically, research on depression biomarkers using EEG recordings revealed high heterogeneity in montage choice and referencing, the choice of electrodes sites to measure asymmetry, the asymmetry formula used and the clinical subtype of depressive pathology. Negligible effect sizes found in meta-analyses also questioned the diagnostic value of fAA in MDD (Gold et al., 2013; van der Vinne et al., 2017). Consequently, more recent approaches have focused on the prognostic value of EEG-derived indicators in depression and affective disorders, more broadly (Kesebir and Yosmaoğlu, 2018; van der Vinne et al., 2019). For our study, we employed methodological decisions aligned with recommended guidelines (Kaiser et al., 2018), in an attempt to limit the inconsistent findings in the discriminative power of EEG alpha asymmetry.

Existing evidence on alpha band in relation to emotional processing suggest recordings from frontal electrode sites have a reduced predictive capability when it comes to discriminating between depressed patients and healthy controls (de Aguiar Neto and Rosa, 2019). To our knowledge, however, little research has been done to examine and compare three homologous electrode sites used to extract alpha band, in relation to depression. Our attempt to compare the most used alpha asymmetry sites (frontal, frontolateral, and parietal) revealed that frontal and frontolateral alpha asymmetry was significantly lower than parietal asymmetry across eyes-open/eyes-closed conditions, for patients diagnosed with depression.

Presence of anxiety (which is often prevalent in MDD) was proposed as being one of the reasons why fAA may not be suitable for diagnostic purposes as anxiety may alter alpha asymmetry and make it more difficult to associate with depression. Correlated dimensions and overlapping symptoms of depression and anxiety raised questions about extent to which shared symptoms can covariate with the alpha asymmetry indices (Horato et al., 2022). In the same line, our results show no significant differences in fAA for patients with comorbid anxiety, even though different underlying neural mechanisms have been described for MDD vs. MADD (Crane et al., 2016).

Apart from comorbid anxiety, our study proposes comparing groups that vary in depression severity and treatment-resistance. The recurrent depressive disorder (RDD) group was contrasted with the major depressive disorder (MDD) and the mixed anxiety-depression disorder (MADD) groups, with no significant differences found in terms of alpha asymmetry, across all sites.

Given the known heterogeneity of depression and the issues raised in differential diagnosis (Ghaemi and Vöhringer, 2011; Fried, 2017; Lorenzo-Luaces et al., 2021), reliable objective criteria such as electroencephalography (EEG) indices would have been helpful in detecting and differentiating depressive states. Currently, there is no consensus in the meta-analytic literature regarding the discriminative power of EEG asymmetry in depression, or in symptoms associated with depression, some highlighting the marker’s ability to discriminate (Bruun et al., 2021), while others concluding inconsistencies, based on existing evidence (van der Vinne et al., 2017; Kaiser et al., 2018; Kołodziej et al., 2021).

Our results confirm the difficulty of finding specific EEG markers with high predictive power (Widge et al., 2019), when there is no research consensus on electrophysiological indices in depression. However, our study is one of very few that directly examined alpha asymmetry in multiple conditions (EO/EC, TRD/non-TRD, RDD/MDD/MADD) and at multiple locations (fAA, flAA, pAA), shedding some light on what could be a promising marker. Presumably, frontolateral alpha asymmetry (flAA) for MADRS-diagnosed depression, and only for the eyes-closed condition, could be considered a candidate. We found a medium association between the flAA and MDD, but an adequate sample size would better clarify the nature of the relationship. Parietal asymmetry might also be a candidate marker; future studies may be able to collect enough evidence in order to provide a cutoff value for clinical discrimination.

The correlation between flAA and MADRS scores, but not BDI II or their composite score, could be explained by the medium correlation between the two diagnostic tools (*r* = 0.47). Our findings raise the issue of convergent validity of the two diagnostic tools, which may further complicate the problem of the many different forms of depression. A deeper investigation of the discrepancy in the assessment of depression of the two instruments would be of interest, as a future direction of research. The lack of significant associations between alpha asymmetry indices and depression is consistent with the more reserved conclusion of recent literature, upon the fAA reliability as a diagnostic marker (Smith et al., 2017; van der Vinne et al., 2017). It may also be in line with the hypothesis of lack of temporal stability of fAA in depressed patients proposed by Debener et al. (2000), who suggested considering the variability of anterior EEG asymmetry (and not its value) as a feature relevant for depression.

Taken together, the results challenge the fAA reliability as marker while proposing further studies on two possible candidate markers (frontolateral alpha asymmetry - for eyes-closed condition - and parietal asymmetry), in an attempt to broaden the understanding of physiological underpinnings of depression. The study is relevant and provides support for future research efforts in the validation of some neurophysiological markers of depression, by advancing some hypotheses that are worth investigating. Also, the study has clinical relevance for neuroregulatory interventions (e.g., neurofeedback), where there are standard protocols targeting alpha asymmetry based on the reliability of the fAA marker. Our findings on flAA and pAA potential might add to efforts of establishing and confirming efficient and reliable protocols for the treatment of depression.

## Data availability statement

The datasets presented in this study can be found in online repositories. The names of the repository/repositories and accession number(s) can be found below: https://osf.io/8vbkf

## Ethics statement

The studies involving human participants were reviewed and approved by Ethics Committee of Clinical Psychiatrical Hospital “dr. Gh. Preda” Sibiu, Romania. The patients/participants provided their written informed consent to participate in this study.

## Author contributions

GM, MB, and CB: study conception and design. GM, A-MR, CB, RF, CT, MR, and AB: data collection. RS-C and GM: analysis and interpretation of results. GM, RS-C, CB, and A-MR: draft manuscript preparation. All authors reviewed the results and approved the final version of the manuscript.

## Funding

This work was supported by the Ministry of Research, Innovation and Digitization through Program 1—Development of the national research-development system, Subprogram 1.2—Institutional performance—Projects for financing excellence in RDI, contract no. 28 PFE/30 December 2021.

## Conflict of interest

The authors declare that the research was conducted in the absence of any commercial or financial relationships that could be construed as a potential conflict of interest.

## Publisher’s note

All claims expressed in this article are solely those of the authors and do not necessarily represent those of their affiliated organizations, or those of the publisher, the editors and the reviewers. Any product that may be evaluated in this article, or claim that may be made by its manufacturer, is not guaranteed or endorsed by the publisher.

## Footnotes

^1^ https://osf.io/8vbkf/
